# Foreign Bodies in the Ear

**Published:** 1875-01

**Authors:** 


Removal of Foreign Bodies from the Ear.—Let the surgeon take six inches, or as much as he pleases—it is always handy and plenty of it—of horse hair, double it into a loop; then having the patient placed on his side, pass the loop into the ear as far as it will go; turn it gently, and at first or second withdrawal the foreign body will come out in the loop. It gives no pain, and cannot do damage.—*The Clinic.*

